# Virtual reality-based training may improve visual memory and some aspects of sustained attention among healthy older adults – preliminary results of a randomized controlled study

**DOI:** 10.1186/s12888-024-05811-2

**Published:** 2024-05-08

**Authors:** Ewa Szczepocka, Łukasz Mokros, Jakub Kaźmierski, Karina Nowakowska, Anna Łucka, Anna Antoszczyk, Javier Oltra-Cucarella, Walter Werzowa, Martin Hellevik, Stavros Skouras, Karsten Bagger

**Affiliations:** 1 Department of Old Age Psychiatry and Psychotic Disorders, Medical University of Lodz, Czechosłowacka 8/10, Lodz, 92-216 Poland; 2 Department of Clinical Pharmacology, Medical University of Lodz, Lodz, Poland; 3 Senopi AG, Zurich, Switzerland; 4 Department of Health Psychology, University Miguel Hernández de Elche, Elche, Spain; 5 The MusicMedicine Consultancy, Vienna, Austria; 6 Stiftelsen CatoSenteret, Son, Norway; 7 Department of Biological and Medical Psychology, University of Bergen, Bergen, Norway

**Keywords:** Virtual reality, Cognitive functions, Visual memory, Sustained attention, Working memory, Head-mounted-display, Older adults

## Abstract

**Background/Aims:**

Older age and cognitive inactivity have been associated with cognitive impairment, which in turn is linked to economic and societal burdens due to the high costs of care, especially for care homes and informal care. Emerging non-pharmacological interventions using new technologies, such as virtual reality (VR) delivered on a head-mounted display (HMD), might offer an alternative to maintain or improve cognition. The study aimed to evaluate the efficacy and safety of a VR-based Digital Therapeutics application for improving cognitive functions among healthy older adults.

**Methods:**

Seventy-two healthy seniors (experimental group *N* = 35, control group *N* = 37), aged 65–85 years, were recruited by the Medical University of Lodz (Poland). Participants were randomly allocated to the experimental group (a VR-based cognitive training which consists of a warm-up module and three tasks, including one-back and dual-N-back) or to the control group (a regular VR headset app only showing nature videos). The exercises are performed in different 360-degree natural environments while listening to a preferred music genre and delivered on a head-mounted display (HMD). The 12-week intervention of 12 min was delivered at least three times per week (36 sessions). Compliance and performance were followed through a web-based application. Primary outcomes included attention and working memory (CNS-Vital Signs computerized cognitive battery). Secondary outcomes comprised other cognitive domains. Mixed linear models were constructed to elucidate the difference in pre- and post-intervention measures between the experimental and control groups.

**Results:**

The users performed, on average, 39.8 sessions (range 1–100), and 60% performed more than 36 sessions. The experimental group achieved higher scores in the visual memory module (B = 7.767, *p* = 0.011) and in the one-back continuous performance test (in terms of correct responses: B = 2.057, *p* = 0.003 and omission errors: B = -1.950, *p* = 0.007) than the control group in the post-test assessment. The results were independent of participants’ sex, age, and years of education. The differences in CNS Vital Signs’ global score, working memory, executive function, reaction time, processing speed, simple and complex attention, verbal memory, cognitive flexibility, motor speed, and psychomotor speed were not statistically significant.

**Conclusions:**

VR-based cognitive training may prove to be a valuable, efficacious, and well-received tool in terms of improving visual memory and some aspect of sustainability of attention among healthy older adults. This is a preliminary analysis based on part of the obtained results to that point. Final conclusions will be drawn after the analysis of the target sample size.

**Trial registration:**

Clinicaltrials.gov ID NCT05369897.

**Supplementary Information:**

The online version contains supplementary material available at 10.1186/s12888-024-05811-2.

## Background/Aims

The world’s population is aging, and the percentage of older people will continue to rise in the coming years. The percentage of people aged 65 or older was 20.8% among the European Union countries in 2021 [1], but by 2050, one in four persons could be 65 years or older in Europe and Northern America [2]. There is an urgent need to enhance the quality and effectiveness of healthcare required by the growing elderly population worldwide. Older age and cognitive inactivity have been associated with cognitive impairment, which in turn is linked to economic and societal burdens due to rising care demands and healthcare costs [3].

Emerging non-pharmacological interventions using new technologies, such as virtual reality (VR), might offer an alternative to maintain or improve cognitive functions among older adults. VR has gained significant attention and potential in various fields and has shown promise in caring for older individuals, whether they have cognitive impairment or not [4]. Cognitive decline is a common concern among older adults and can significantly impact daily functioning and quality of life. Finding effective interventions to enhance cognitive functions can help mitigate the negative consequences of cognitive decline and promote healthy aging [5].

Virtual reality (VR) encompasses various computer-generated environments that provide digital experiences. The intensity and quality of immersion in these virtual worlds differentiate the main types of VR. Non-immersive VR is the most common type, where individuals interact with virtual worlds through devices like computers, tablets, or smartphones. They remain aware of their physical surroundings while engaging with the virtual content [6]. Fully immersive VR provides a complete sense of presence in the virtual world. Specialized hardware, such as head-mounted displays (HMDs) and bodysuits, eliminates sensory input from the real world, creating the illusion of being in the virtual environment [7]. Augmented reality (AR) overlays virtual elements in the real world, enhancing the perception of physical objects with computer-generated information. A practical way to augment reality is through the visual system using hands-free wearables, such as smart glasses. In augmented reality, the user can see the components of the virtual world but cannot interact with them [8]. Mixed reality (MR) is a form of hybrid reality in which the real and virtual elements can interact with one another, thereby granting the user the ability to interact with both real and virtual objects [9]. Extended reality (XR) is a broad term encompassing all immersive technologies, including AR, VR, and MR, as well as future technologies yet to be developed [10].

VR’s immersive and interactive nature provides unique opportunities for enhancing cognitive functions. Research has demonstrated that VR can enhance cognitive performance and promote neuronal plasticity, increasing cortical grey matter volumes and a higher concentration of electroencephalographic beta waves [10]. In the context of neurorehabilitation, VR has been utilized to assist patients with stroke [11] or traumatic brain injury [12] in their recovery process and may even be an essential ingredient for the replacement of lost functions through an appropriate brain-computer interface (BCI) that controls robotic devices [13]. For older adults experiencing cognitive decline and social isolation, VR can offer therapeutic interventions targeting various cognitive domains, such as memory, attention, executive functions, and enhance social engagement [14]. The use of VR-based digital therapeutics has the potential to provide accessible and engaging interventions for cognitive enhancement. Traditional cognitive training methods may be limited in terms of accessibility, motivation, and ecological validity. The research by Zhong et al. [15] supports the idea that VR interventions are considered a cost-effective, accessible, flexible, and comprehensive option for individuals who may face challenges attending outpatient appointments due to factors such as distance, lack of transportation, or disability. This suggests that traditional face-to-face interventions may not always be accessible to all individuals, highlighting the importance of shifting care from clinical settings to patients’ homes to improve accessibility and effectiveness in cognitive training programs. By utilizing VR technology, which provides immersive and engaging experiences, individuals can potentially overcome barriers related to accessibility and motivation that may be associated with traditional cognitive training methods.VR technology offers a unique opportunity to create immersive and interactive experiences that can simulate real-world scenarios and engage multiple sensory modalities. VR interventions can enhance the effectiveness and engagement of cognitive interventions, leading to better outcomes for older adults [16].

The use of VR in healthcare settings, particularly in mental health, has been explored [17]. VR technology has been investigated for cognitive enhancement in various contexts, such as stroke rehabilitation and psychotherapy, highlighting its versatility and potential benefits [18]. Virtual reality-based interventions have also been shown to improve cognitive function in individuals with neurocognitive disorders, suggesting a broader applicability beyond physical rehabilitation [19]. Additionally, VR interventions have been found to selectively enhance cognitive performance in healthy older adults, indicating the potential for targeted cognitive training using VR technology [5]. While the literature on VR interventions in healthcare is expanding, its application for cognitive enhancement in healthy older adults who can use VR at home is still an emerging area of research [20]. A range of studies have demonstrated the potential of home-based cognitive VR interventions for healthy older adults. Gamito [21] found that VR-based cognitive stimulation led to improvements in general cognition, executive functioning, attention, and visual memory. Similarly, Zając-Lamparska [22] reported positive changes in cognitive functioning in healthy older adults after VR-based cognitive training. Basak [23] highlighted the benefits of virtual cognitive training for episodic memory and executive functions in both healthy aging and mild cognitive impairment. Moreover, Thapa [24] further supported these findings, showing that a VR intervention program improved executive function, brain function, and physical function in older adults with mild cognitive impairment. These studies collectively suggest that home-based cognitive VR interventions can be effective in enhancing cognitive and physical function in healthy older adults. Understanding home-based VR interventions’ benefits and potential limitations is crucial for developing evidence-based strategies to support healthy aging and cognitive well-being [5]. Further research is still needed to assess the impact of VR interventions on specific cognitive domains and identify the optimal protocols for therapeutic use.

Existing products and services may be considered too expensive for the home end user and limited to clinics, nursing homes and rehabilitation centers [25]. In the global phenomenon of aging societies, the potential of prevention as the most efficient strategy for sustainable healthcare is evident. Therefore, providing seniors with a home-based service that offers scientifically validated, safe, engaging, and personalized content to enhance cognitive abilities would benefit the overall population. The study aimed to evaluate the efficacy of a VR-based Digital Therapeutics application for improving cognitive functions among healthy older adults. The neurocognitive clinical evaluation will include the following domains: composite memory, verbal memory, visual memory, psychomotor speed, reaction time, complex attention, cognitive flexibility, processing speed, executive function, simple attention, sustained attention, working memory and motor speed. The acceptance of VR technology among older adults will also be investigated.

## Methods

The CoSoPhy FX study was designed as a randomized, parallel-group, two-arm, superiority study with an aimed 1:1 allocation ratio. Therefore, the study was conducted with reference to the CONSORT 2010 Statement: updated guidelines for reporting parallel group randomised trials [26]. The study protocol was registered with Clinicaltrials.gov (ID NCT05369897) on 11/05/2022. Also, the full and detailed study protocol has been published [27].

The protocol and the template informed consent form received approval from the Bioethical Committee at the Medical University of Lodz, Poland (RNN/222/21/KE). Informed consent was obtained from all subjects and/or their legal guardian(s). The Medical University of Lodz (Poland) was responsible for recruiting high-functioning seniors aged 65–85 from a community-dwelling setting. High-functioning seniors were defined as being over the age of 65 years and maintaining their functional independence concerning activities of daily living, including the ability to go on a long walk and the ability to interact with standard modern technology (e.g., using a smartphone to send a message). The study, encompassing recruitment and completion of follow-up, was scheduled to run from January 2022 to May 2023. The recruitment phase itself extended from January 2022 to mid-January 2023. A research team member introduced the study to participants and performed an initial demonstration of the equipment used for the intervention. Subsequently, the participants had an opportunity to ask any remaining questions, and afterwards, they signed the informed consent form. The eligibility criteria are presented in Table 1.

**Table 1:** Inclusion and exclusion criteria

Inclusion criteria	Exclusion criteria
1. Individuals aged 65–85 years old	1. Neuropsychiatric disorders (MoCA < 26 points)
2. Stable medical condition (e.g., well-controlled diabetes or hypertension)	2. Abuse or addiction to alcohol, drugs, and tranquillizers (DSM-5)
3. Undisturbed locomotion	3. Blurred vision that cannot be corrected with lenses or glasses
4. Independent in everyday functioning	4. Auditory pathologies causing significant hearing loss
5. Capable of going on long walks without assistance	5. High sensitivity to motion sickness
6. Able to use standard modern technology	6. Migraines
	7. Epilepsy
	8. Obesity (BMI > 30 kg/m^2^)
	9. Deemed unsuitable for participation by the investigator due to reasons exceeding the bespoken exclusion criteria, e.g. particular comorbidities or specific results of diagnostic tests

*MoCA* Montreal Cognitive Assessment, *BMI* Body Mass Index

A clinical assessment was conducted using the Montreal Cognitive Assessment (MoCA) scale [28] to exclude participants with suspected cognitive impairment (i.e., achieving a total score lower than 26 points). *N* = 310 subjects were screened for eligibility, and *N* = 122 were excluded because they did not meet the inclusion criteria. The most common cause of exclusion was the MoCA score below 26 points, suggesting the presence of objective cognitive impairment. Discrepancies in the assumed MoCA’s cut-off for cognitive impairment may vary depending on race, ethnicity, and education level [29, 30]. We are aware that a high cut-off poses a risk of false positive decisions regarding the exclusions of participants. Yet, we decided to keep the cut-off of 26 to ensure high sensitivity, in accordance with previous studies [31]. The recruitment rate (61%) was acceptable. After the participants had been screened for eligibility, the research team produced a list of eligible participants. Participants were randomly allocated to an experimental or control group with a 1:1 allocation using a computerized random number generator (random.org) [32]. A simple randomization method was adopted. One author (ESz) generated the sequences of numbers from 1 to 200 and assigned them to the intervention based on the following assumption: 1–100 ~ experimental group, 101–200 ~ control group. Another author (AA) assigned the participants to either the experimental or control group according to the list of randomized numbers. To ensure concealment, the list was kept by an independent research team member (JK) who did not participate in the subject recruitment process. No other stratification was utilized.

**Fig. 1:**
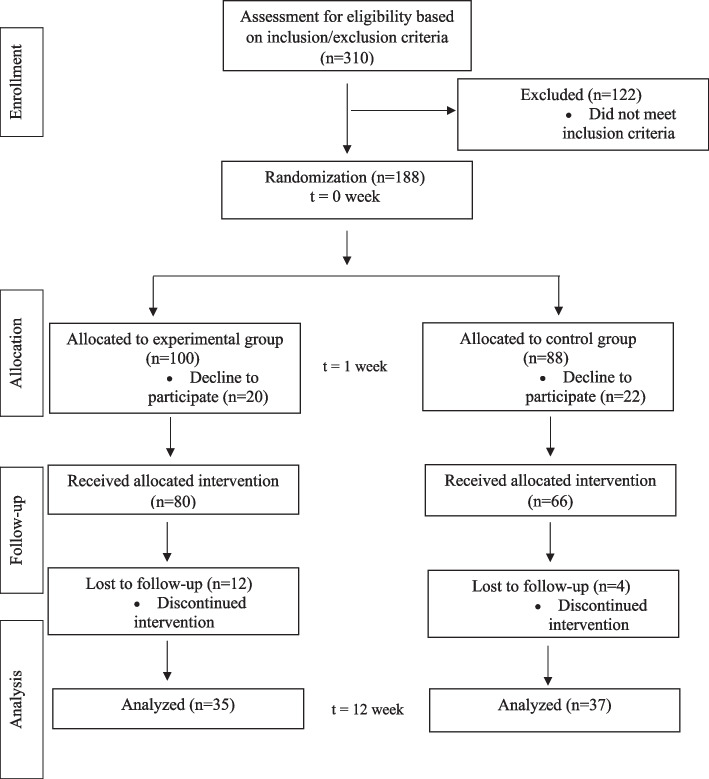
CoSoPhy flowchart

The target for our study was to have 200 participants, evenly distributed with 100 in each group. However, 310 individuals participated in the initial screening process during the recruitment phase. Due to our inclusion criteria and the sequential nature of recruitment, only 188 individuals were deemed eligible and thus included in the study by the time recruitment was concluded. This resulted in an uneven distribution of participants between the groups, with *N* = 100 allocated to the experimental group and *N* = 88 to the control group, giving an allocation ratio of approximately 1:0.88.In the experimental group, *N* = 20 participants resigned before the start of the intervention, and *N* = 12 withdrew during the intervention due to somatic and mental health issues unrelated to the hereby study. In the control group, the respective numbers were *N* = 22 and *N* = 4. In this preliminary analysis, we assessed *N* = 72 high-functioning seniors (experimental group *N* = 35, control group *N* = 37). The participant flowchart is presented in Fig. 1.

The characteristics of the studied group are presented in Table 2.

**Table 2:** Characteristics of the studied group of healthy older adults

	**EG (*N* = 35)**	**CG (*N* = 37)**
Age, M ± SD	69.3 ± 2.84	71.3 ± 4.2
School years, M ± SD	15.3 ± 2.6	15.8 ± 3.0
Sex, N (%)		
*Male*	10 (29%)	8 (22%)
*Female*	25 (71%)	29 (78%)
Education level, N (%)		
*Secondary*	8 (23%)	7 (19%)
*Vocational*	4 (11%)	5 (14%)
*Higher*	23 (66%)	25 (68%)
Chronic diseases, N (%)	31 (89%)	84 (84%)
Place of residence, N (%)		
*City*	32 (91%)	34 (92%)
*Rural area*	3 (9%)	2 (5%)
*Did not specify*	0 (0%)	1 (3%)
Marital status, N (%)		
*Single*	2 (6%)	5 (14%)
*Married*	19 (54%)	18 (49%)
*Informal relationship*	0 (0%)	2 (5%)
*Widowed*	10 (29%)	6 (16)
*Divorced*	4 (11%)	6 (16%)
With whom live, N(%)		
*Alone*	14 (40%)	22 (59%)
*With family*	21 (60%)	15 (41%)
MoCA total score, N ± SD	27.7 ± 1.1	27.7 ± 1.5

*EG* experimental group, *CG* control group, *M* mean, *SD* standard deviation, *N* number of observations, *%* percentage, *MoCA* Montreal Cognitive Assessment test

The intervention was delivered using a VR-HMD worn by the seniors (Pico Neo 3 Pro in the experimental group and Pico G2 in the control group). The content shown in the VR-HMD consists of two key components:

1. Base content: The base consent comprises high-quality 360-degree photographs and videos from natural environments (from the real world), such as a relaxing mountain environment.
2. Graphical overlay: The base content has a graphical overlay that has two key components:
  i) Hands: the user has to hold a controller in each hand, enabling them to see their hands positioning inside the VR environment (“virtual hands”).
  ii) Graphical objects: e.g., balloons that users can grab with their virtual hands, appear inside the VR environment.

The intentions of this were:

i) to motivate the user to perform physical activities with their hands (moving their arms and upper body will be required to reach for graphical objects); and
ii) to perform cognitive exercises while using their arms to reach out for specific graphical objects according to the rules of each cognitive task.

During the training, the participants could also listen to a selection of musical pieces from the HealthTunes database [33] (the music selection is presented in Supplementary Table S1).

In the experimental group, the cognitive training consisted of the following parts:

1. Warm-up: a simple exercise to familiarize the participant with the task.
2. Focus: an exercise of focused attention in which participants must select balloons that match the color of their hands.
3. Switch: an exercise of alternating attention in which participants must reach for shapes alternately with the matching hand color.
4. Memory: an exercise of working memory based on n-back tasks [34, 35] in which participants must tap a balloon the same color as n-balloons earlier.

Participants in the control group passively experienced 360-degree photographs and videos from natural environments without cognitive training. This technique is frequently employed in cognitive studies, offering a passive experience devoid of cognitive challenges [36]. Regardless of the group, the participants were encouraged to use the VR-HMD a minimum of three times weekly for three months (36 sessions total, each lasting 12 min). The first training session occurred at the Medical University of Lodz, where participants were trained to use the VR-HMD. At the end of the session, participants took the VR-HMD to their homes, where they could use the VR-HMD at any time and any place, but it was recommended that they sit on an armchair or a chair with armrests to minimize the risk of falling. Additionally, participants were provided with a comprehensive user manual that detailed the operation and maintenance of the VR-HMD. In the event of questions or technical difficulties, participants had the opportunity to consult an IT specialist, who was an integral member of the research team, thereby ensuring ongoing support throughout the study period.

At the end of the intervention, the satisfaction questionnaire was used to collect feedback, including information on perceived side effects. Compliance and performance were followed through a web-based application.

Cognitive outcome testing was performed at baseline and after the intervention using the CNS Vital Signs (CNSVS) is a computerized neurocognitive test battery developed as a routine clinical screening instrument [37]. The CNS-VS tool has been validated through rigorous research and testing. Studies have demonstrated its reliability and validity in assessing cognitive function across different populations. The tool has shown sensitivity in detecting subtle cognitive deficits and progressive decline or improvement. It has been used in various clinical settings to aid in treatment planning and monitor cognitive outcomes [38]. It comprises eight tests: verbal memory (VBM), visual memory (VSM), finger tapping (FTT), symbol digit coding (SDC), Stroop Test (ST), shifting attention test (SAT), continuous performance test (CPT), and four-part continuous performance test (FPCPT). A result includes neurocognitive clinical evaluation domains:

- composite memory (measures how well the subject can recognize, remember, and retrieve words and geometric figures);
- verbal memory (measures how well the subject can recognize, remember, and retrieve words);
- visual memory (measures how well the subject can recognize, remember and retrieve geometric figures);
- psychomotor speed (measures how well the subject perceives, attends, responds to complex visual-perceptual information and performs simple fine motor coordination);
- reaction time (measures how quickly the subject can react, in milliseconds, to a simple and increasingly complex direction set);
- complex attention (measures the ability to track and respond to a variety of stimuli over lengthy periods and/or perform complex mental tasks requiring vigilance quickly and accurately);
- cognitive flexibility (measures how well the subject is able to adapt to rapidly changing and increasingly complex set of directions and/or to manipulate the information);
- processing speed (measures how well a subject recognizes and processes information, i.e., perceiving, attending/responding to incoming information, motor speed, fine motor coordination, and visual-perceptual ability);
- executive function (measures how well a subject recognizes rules, categories and manages or navigates rapid decision-making);
- simple attention (measures the ability to track and respond to a single defined stimulus over lengthy periods while performing vigilance and response inhibition quickly and accurately to a simple task);
- sustained attention (measures how well a subject can direct and focus cognitive activity on specific stimuli),
- working memory (measures how well a subject can perceive and attend to symbols using short-term memory processes), and
- motor speed (measures the ability to perform simple movements to produce and satisfy an intention towards a manual action and goal) [37].

The participants were subjected to health insurance throughout the intervention, covering any harm according to the insurance policy.

### Statistical methodology

The primary analysis involved *N* = 72 high-functioning seniors (experimental group *N* = 35, control group *N* = 37). The statistical analysis was performed with the jamovi software, version 2.2 (the jamovi project, retrieved from https://www.jamovi.org). The continuous variables were characterized by means with standard deviations, and the categorical variables – by a number of observations with the proportion (percentage) from the whole. A statistically significant interaction between the two factors (group * timepoint) for the cognitive performance scores was assumed to be an indicator of the improvement after the VR training intervention (compared to before the intervention) only for the experimental group (but not for the control group). Thus, a linear mixed model was constructed for each of the cognition-associated variables. Each model was controlled for age, sex, and the number of school years of the participants. Marginal coefficient of determination (R^2^) was reported for each model to indicate the effect size attributed to the fixed factors. Since this is a preliminary analysis, no calculation of the sample size nor type 1 and type 2 error corrections were performed. The significance level was adopted for α = 0.05.

## Results

In the case of the cognitive domains, the tested fixed effect of a group*timepoint interaction was statistically significant in the case of visual memory in favor of the experimental group after the intervention (B = 7.767, *p* = 0.011). The effect was independent of age, sex, and the number of school years. The interaction was not statistically significant for the remaining cognitive domains (Table 3).

**Table 3:** Fixed effect parameters of a group*timepoint interaction (as a measure of improvement during the virtual reality-based training in the experimental versus control group) for each of the assessed cognitive domains in the studied group of healthy older adults

Cognitive domain	R^2^	B	SE	t	*p*
**Visual memory**	**0.081**	**7.767**	**2.980**	**2.607**	**0.011**
Verbal memory	0.075	0.670	1.238	0.541	0.590
Sustained attention	0.109	2.133	2.035	1.048	0.298
Simple attention	0.020	1.312	1.265	1.037	0.303
Complex attention	0.128	-0.904	2.032	-0.445	0.658
Working memory	0.166	1.073	1.259	0.852	0.397
Executive function	0.209	1.233	3.355	0.367	0.714
Cognitive flexibility	0.208	0.655	3.406	0.195	0.846
Reaction time	0.123	35.22	33.700	1.045	0.300
Processing speed	0.102	-6.080	5.249	-1.158	0.251
Motor speed	0.089	3.456	5.740	0.602	0.549
Psychomotor speed	0.127	8.948	7.583	1.180	0.242

Each model was controlled for sex, age, and number of school years. R^2^ – marginal coefficient of determination, B – unstandardized fixed effect parameter in the model, SE – standard error of the B parameter, t – statistics in the t-test, p – probability in the test, values in bold - statistically significant differences highlighted

The results of the neurocognitive tests measuring visual memory, working memory, and attention were performed in greater detail. Here, the group*timepoint interaction was significant for the number of immediate correct hits in the visual memory test – an increase in the score was observed for the experimental group in the post-intervention period (B = 2.931, *p* < 0.001). Also, the interaction was significant for the subscores of the one-back task (part 3 of the four-part continuous performance test), namely the number of correct responses (B = 2.057, *p* = 0.001), and the number of omission errors (B = -1.950, *p* = 0.007). See Table 4 for detailed results.

**Table 4:** Fixed effect parameters of a group*timepoint interaction (as a measure of improvement due to the virtual reality-based training in the experimental versus control group) for the results of the neurocognitive battery tests assessing the visual memory, attention and working memory

Test name	Subscores	R^2^	B	SE	t	*p*
**Visual memory test**	Correct hits - delay	0.043	0.903	0.574	1.572	0.121
	**Correct hits - immediate**	**0.082**	**2.931**	**0.846**	**3.464**	** < 0.001**
Stroop test	Simple reaction time	0.147	-0.309	32.410	-0.010	0.992
	Complex reaction time	0.114	47.680	40.390	1.181	0.242
	Reaction time correct	0.111	21.99	38.190	0.576	0.567
Continuous Performance Test	Correct responses	0.016	1.115	1.189	0.938	0.352
	Omission errors	0.024	0.505	2.033	0.248	0.804
	Commission errors	0.051	1.426	1.675	0.851	0.396
**Four-Part Continuous Performance Test**	Part 1 average correct reaction time	0.038	33.92	38.59	0.879	0.383
	Part 2 correct responses	0.020	0.374	0.339	1.102	0.274
	Part 2 average correct reaction time	0.060	36.27	28.67	1.265	0.208
	Part 2 incorrect responses	0.080	1.225	0.896	1.367	0.174
	Part 2 average incorrect response time	0.026	-43.922	57.710	-0.761	0.449
	Part 2 omission errors	0.020	-0.374	0.339	-1.102	0.274
	**Part 3 correct responses**	**0.093**	**2.057**	**0.659**	**3.121**	**0.003**
	Part 3 average correct reaction time	0.054	50.17	27.69	1.812	0.074
	Part 3 incorrect responses	0.013	0.450	0.650	0.693	0.491
	Part 3 average incorrect response time	0.021	-120.37	97.040	-1.240	0.217
	**Part 3 omission errors**	**0.086**	**-1.950**	**0.697**	**-2.799**	**0.007**
	Part 4 correct responses	0.129	1.262	0.972	1.299	0.198
	Part 4 average correct reaction time	0.012	23.209	44.610	0.520	0.605
	Part 4 incorrect responses	0.050	-0.019	1.109	-0.018	0.986
	Part 4 Average incorrect response time	0.045	57.350	101.360	0.566	0.573
	Part 4 Omission errors	0.129	-1.262	0.972	-1.299	0.198

Each model was controlled for sex, age, and number of school years. R^2^ – marginal coefficient of determination, B – unstandardized fixed effect parameter in the model, SE – standard error of the B parameter, t – statistics in the t-test, p – probability in the test, values in bold - statistically significant differences highlighted

## Discussion

### Principal results

In hereby preliminary analysis, it was observed that a specific immersive VR-based cognitive training may improve at least some aspects of visual memory, sustained attention and working memory. A favorable effect of the intervention was seen in the case of the scores of visual memory (correct hits-immediate) and the one-back task (the number of correct responses and omission errors), evaluating working memory and sustained attention.

### Comparison with prior work

Cognitive training systems aim to improve specific domains or global cognition by engaging users in cognitively demanding tasks. A relatively new research area of growing interest in cognitive interventions concerns the use of VR, which creates a simulated environment that mimics a real or imaginary setting, allowing users to feel as if they are physically present and engage their senses within that virtual world [39]. VR immersion levels can be categorized as low, moderate, or high. VR-HMD, which was used in this study, falls under the high immersion category, which involves stimulating more than two sensory modalities (e.g., vision, hearing, proprioception, and motor skills) with spatially oriented stimuli. Opting for a higher level of immersion is recommended as it can enhance the patient’s sense of presence, leading to more pronounced behavioral responses [40].

The potential of immersive VR for cognitive training in the elderly population has gained significant attention in recent years. Meta-analyzes and systematic reviews provide evidence for the efficacy of this type of intervention in various populations, including healthy older adults as well as individuals with mild cognitive impairment (MCI), dementia, and traumatic brain injury [4, 17, 41, 42].

In the present study, we found a significant group*timepoint interaction for visual memory score (correct hits immediate), which suggests that the VR intervention had a positive impact on visual memory performance. This finding is particularly noteworthy as visual memory plays a crucial role in higher cognitive processes [43].

Our finding regarding the visual memory domain improvement aligns with previous research evaluating the effectiveness of VR cognitive interventions among healthy older adults [21, 44]. In the study published by Gamito et al., twenty-five participants, aged 65–85, underwent 12 VR training sessions between the pre-treatment and post-treatment assessments. A significant increase was seen between the two assessments for some neuropsychological measures: visual memory, attention, and cognitive flexibility [44]. In the subsequent study, forty-three healthy older adults were divided into two groups: an experimental group underwent a VR-based cognitive stimulation (two 30-min sessions per week for six weeks), and an active control group underwent a paper-and-pencil cognitive stimulation. The outcomes were assessed at the baseline and after intervention by well-established cognitive and executive functioning tests (the Frontal Assessment Battery—FAB, the Wechsler Memory Scale-Revised—WMS, the Rey Complex Figure—RCF and the d2 test). The results suggested the positive effects of VR cognitive stimulation on visual memory, attention, executive function, and general cognition [21].

The significant group*timepoint interaction for the subscores of the one-back task, namely the number of correct responses and the number of omission errors, may indicate a positive effect of the VR intervention on working memory and attention. However, no significant effect was seen in the case of working memory and attention CNS-VS scores. Working memory is a cognitive function that is crucial for various daily activities, such as learning, problem-solving, and decision-making. It involves the temporary storage and manipulation of information, while attention is crucial for maintaining focus and selectively processing relevant stimuli [45]. Age-related decline in working memory has been well-documented, with older adults exhibiting deficits in this cognitive domain compared to their younger counterparts [46, 47]. Therefore, interventions enhancing working memory performance in older adults are of great interest.

The current study found a significant group*timepoint interaction for the number of correct responses in the one-back task. This finding suggests that the VR intervention may have a positive impact on the participants’ working memory performance. However, it was not confirmed for a combined CNS-VS working memory score. The improvement in the number of correct responses indicates enhanced accuracy and efficiency in retrieving and manipulating information in working memory [48]. This finding aligns with previous studies that have reported improvements in working memory performance following VR interventions [24, 49].

Furthermore, the significant group*timepoint interaction for the number of omission errors in the one-back task provides additional support for the positive effects of the VR intervention on attention. Omission errors, also known as false negatives, occur when participants fail to respond to a target stimulus. They reflect lapses in attention and can be indicative of attentional deficits [50]. The reduction in omission errors observed in the VR intervention group suggests that the intervention enhanced attentional processes, leading to improved detection and response to target stimuli. This finding is consistent with previous research showing that VR interventions can enhance attentional performance [51, 52].

However, it is necessary to note that our results were not statistically significant for the remaining cognitive domains assessed in the CNS-Vital Signs battery. This may suggest that the VR-based cognitive training program may hold a limited effect on other specific cognitive functions, such as processing speed, executive function, and verbal memory. However, previous meta-analyses reported no positive effects on memory [15, 53, 54], execution function [53], and attention [15, 53] among patients with MCI.

The heterogeneity of the study designs, target groups (healthy older adults vs MCI and dementia), and outcome measures may contribute to the observed discrepancies.

The lack of significant effects on remaining cognitive domains may be attributed to various factors, such as individual differences in baseline cognitive abilities, the duration and intensity of the intervention, and potential ceiling effects. The current study delivered the interventions at least three times per week for 12 min per session for 12 weeks. This frequency and duration were chosen based on previous studies that have shown positive effects of VR interventions on cognitive function in older adults [55]. However, it is worth noting that the frequency and duration of VR-based cognitive interventions can vary considerably between studies. The duration of a single intervention ranged from 10 to 50 min [24, 56], the number of interventions ranged from 10 to 40 sessions, and the course of the intervention varied from 3 to 12 weeks [57]. These variations in frequency and duration highlight the lack of standardized guidelines for VR interventions in cognitive training. Future studies could explore varying intensities and durations of the intervention in more detail to better understand the mechanisms underlying the observed effects. Additionally, further studies should investigate the impact of the VR intervention on populations with mild cognitive impairment to understand its broader applicability and effectiveness better.

Despite heterogeneity in study populations and methodological differences in previous studies, our work provides further evidence to support the benefits of immersive VR cognitive training in eliciting improvements in visual memory, working memory, and sustained attention among healthy older adults.

The degree to which participants have been exposed to and accept new technologies may play a crucial role in influencing the outcomes of a study examining the effects of virtual reality (VR) cognitive training on healthy individuals over the age of 65. This is because their prior experience and comfort level with technology can impact how they interact with VR, potentially affecting the study’s findings.

Participants with higher exposure to technology might have a shorter learning curve when it comes to using VR equipment and applications. This can result in quicker adaptation to the VR training environment, potentially leading to more immediate improvements in cognitive tests conducted within the VR setting. Conversely, individuals with less exposure may require additional time to become comfortable with the technology, which could initially hinder their performance or progress in the study [58].

Acceptance of new technologies is closely linked to a participant’s motivation and engagement level. Those who are more open and positive towards using new technologies might be more motivated to engage with the VR cognitive training, thereby potentially benefiting more from the intervention. On the other hand, participants who are apprehensive or sceptical about new technologies might not engage with the training as deeply, which could affect their outcomes [59].

Participants with greater technological proficiency gained through regular interaction with various forms of technology may find navigating VR interfaces and understanding instructions within the VR environment more intuitive. This proficiency can influence the effectiveness of the training, as these participants might be able to focus more on the cognitive tasks rather than the operation of the VR system itself [60].

Technological exposure can indirectly influence participants’ initial level of cognitive function. Regular use of technology has been associated with certain cognitive benefits, such as improved problem-solving skills and better memory recall. Thus, participants who frequently use technology might already have a higher baseline in some cognitive domains, which could affect the perceived impact of VR cognitive training [61, 62].

Acceptance of technology also influences how participants perceive and report adverse effects. Those with less exposure to VR and technology might report higher levels of discomfort, dizziness, or disorientation—commonly known as cybersickness—than their more tech-savvy counterparts. This difference in the experience of adverse effects could impact participants’ willingness to continue with the training and their overall assessment of its efficacy [63].

In our study, participants were asked about their use of new technologies and to what extent they engaged with them during the recruitment phase. Specifically, 26% of participants learned about the study through a social media post, indicating their engagement with digital platforms. Regarding technology use, 45% of the participants reported using a smartphone and a computer, 18% used these devices along with a smartwatch or smart band, and 3% also included an e-book reader in their technology repertoire. Moreover, to assess participants’ attitudes towards new technologies, we asked if they were scared to use new technologies, with 26% affirming, and whether they found new technologies easy to use, with 69% agreeing. This information helps us understand the technology exposure and acceptance level among participants.

To summarize, the level of exposure and acceptance of new technologies among older adults can influence various aspects of a study on VR cognitive training, from initial adaptation to the technology to engagement levels and the reporting of adverse effects. These factors are crucial for researchers to consider when designing the study, interpreting its results, and generalizing the findings to broader populations.

### Limitations

The results of the present study contribute to the existing literature on the relationship between cognitive training and cognitive performance. The present study has several strengths, including the use of a randomized controlled design, a relatively large sample size, and objective measures of cognitive performance. However, there are also some limitations that should be considered.

Firstly, only some cognitive variables improved (visual memory, working memory and sustained attention). It is necessary to note that our results were not statistically significant for the remaining cognitive domains assessed in the CNS-Vital Signs battery. This may suggest that the VR-based cognitive training program may hold a limited effect on other specific cognitive functions.

Secondly, the study sample consisted of healthy seniors, which may limit the generalizability of the findings to older adults with cognitive impairments or other health conditions. Future research could investigate the effects of VR-based cognitive training in more diverse populations.

Thirdly, the intervention duration of 12 weeks and intensity (at least three times per week, each session lasting for 12 min) may not have been sufficient to capture the full extent of cognitive improvements that could be achieved with longer-term interventions. Additionally, the absence of a post-intervention follow-up limits our understanding of the long-term durability of the cognitive improvements observed. Future studies could explore the effects of extended interventions on cognitive outcomes.

In this study, our primary focus was to explore the potential impacts of VR cognitive training on healthy individuals over the age of 65. While the unique contributions of VR to cognitive enhancement were highlighted, we acknowledge the absence of a direct comparative analysis with other forms of cognitive training, including non-digital methods and alternative virtual reality applications. This limitation was primarily due to the initial scope of the research, which was designed to assess the feasibility and initial effectiveness of a specific VR intervention without a comparative framework. Additionally, logistical and resource constraints limited our ability to incorporate multiple intervention groups.

However, we recognize the importance of comparative effectiveness research in fully understanding the relative benefits and applicability of different cognitive training methods. Future research could benefit from including a broader range of comparative interventions to provide a more comprehensive evaluation of VR cognitive training’s efficacy relative to other approaches. Such studies would not only offer valuable insights into the most effective cognitive training methods for older people but also help tailor interventions to meet diverse needs and preferences.

## Conclusions

In conclusion, the present study supports the growing body of literature on the potential of immersive VR-based training as a non-pharmacological intervention to improve such cognitive domains like visual memory, working memory, and sustained attention. Immersive VR holds great promise as a flexible and personalized training environment for cognitive training in older adults, with the potential to improve cognition, autonomy, and overall quality of life. Further research is needed to replicate outcomes and standardize VR intervention protocols before widespread implementation in standard care.

### Supplementary Information

Supplementary Material 1.

## Data Availability

The datasets used and/or analysed during the current study are available from the corresponding author upon reasonable request.

## Abbreviations

BMI: Body Mass Index
DSM-5: Diagnostic and Statistical Manual of Mental Disorders
HMD: Head-mounted display
MCI: Mild cognitive impairment
MoCA: Montreal Cognitive Assessment
VR: Virtual reality
