# Supplementary material for: Virtual reality-based training may improve visual memory and some aspects of sustained attention among healthy older adults – preliminary results of a randomized controlled study
Source: BMC Psychiatry. 2024 May 8;24:347. doi: 10.1186/s12888-024-05811-2 (PMC11080129; doi:10.1186/s12888-024-05811-2)
Supplement: Supplementary file 1 — Supplementary Material 1. [file 12888_2024_5811_MOESM1_ESM.docx]

**Table 1. Music selection.**

| **Classical** | **Ambient** | **Electronic** | **Jazz** | **Rock** |
| --- | --- | --- | --- | --- |
| **Opera:**  La Traviata  Un ballo in Maschera  Ernani(Preludio)  Aida  Don Giovanni Ouverture  Le Nozze di Figaro Ouverture  Macbeth (Preludio)  Luisa Miller  Nabucco  Rigoletto (Preludio)  Zauberfloete Ouverture  **Symphony/Concerti:**  Poco Allegro  Concerto RV 90 In D Major_ II. Largo  Concerto No 6 - ii Allegro  Concerto in G minor for recorder _La notte_, RV 439 op. 10 No. 2 Presto  III Allegro  Concerto in D major for violin _Grosso Mogul_, RV 208 Allegro  Concerto MWN IV 7 - 1. Allegro  Concerto RV 362 In B-Flat Major, Op. 8, No. 10_ I. Allegro  Concerto RV 362 In B-Flat Major, Op. 8, No. 10_ III. Allegro  Concerto No 2 - iv Allegro  Concerto RV 151 In G Major_ III. Allegro  Suite in D minor - 4. March Bourree  Il Corsaro  Un ballo in maschera | Indian Yellow  Luminous  Natural Light | Technotic  Techno Pop  Technotic  Touchstone  Out Of Space (Techno Underworld Remix)  Sure Victory  Techno Bone  Get Them  Groovacious  I Love Techno  Burn with Me (Victoria Edit)  Change This  Closer (Techno Mix)  Blackmaster - Club Confidential  Dial M For Moguai - King Of Rock (DJ Moguai Mix)  Stone Factory - Sunset  Baby Doc feat. S-J - Back To Love  Blackmaster - Club Confidential  Stone Factory - Sunset  Change This  I Love Techno  Sure Victory  Groovacious  Techno Bone  Touchstone  Get Them  Groovacious  Sure Victory  Change This  Groovacious | Hindustan  S'Wonderful  Singin' In The Rain  Soul Bossa Nova  On The Street Where You Live  One Note Samba  Rag Mop  Joshua  Just One Of Those Things  Lalo Bossa Nova  Leap Frog  Mexican Hat Dance  On The Beach At Waikiki  Desafinado  I've Got My Love To Keep Me Warm  I've Got The Sun In The Morning  (True) You Don't Love Me [Voice]  Bill Bailey (Won't You Please Come Home)  Bourbon Street Parade (Barbarin) Commodore  Careless Love  Dixie  Do Lord  Down by the Riverside  Eyes of Texas  Georgia Camp  His Eye Is On the Sparrow  Hot Time in the Old Town Tonight  Ain't Got Nobody  Georgia On My Mind  God Will Take Care of You | Satellite Of Love  California  Carey  Do I Ever Cross Your Mind (w_ Dolly Parton)  Dogsong aka Sleep Dog Lullaby  Draft Daughter's Blues aka Ootischenia  Girl from the North Country  Grassy Grass Grass  In My Time Of Dying  Little Black Bear  Little Green  Little Seed  Little Sugar  Merry-Go-Round  My dolly  One Day Old  Only In The Past  Rain And Snow  Love's A Mystery  Lovers' Eyes  Mat Devine feat Suzy - 03 - California Love Song  Love Walks In  Love Will Bring Us Back Together  Love  She Loves You  VanHalenWhenItsLove  You Need Love Like I Do  My Love Is My Love  Rhythm Of Love |
